# Inborn Errors of Immunity and Their Phenocopies: CTLA4 and PD-1

**DOI:** 10.3389/fimmu.2021.806043

**Published:** 2022-01-28

**Authors:** Yuwei Hao, Matthew C. Cook

**Affiliations:** ^1^ Centre for Personalised Immunology and Department of Immunity and Infectious Diseases, John Curtin School of Medical Research, Australian National University, Acton, ACT, Australia; ^2^ Department of Immunology, Canberra Hospital, Woden, ACT, Australia

**Keywords:** CTLA4, PD-1, immune checkpoint inhibitor, phenocopy, immune deficiency

## Abstract

Elucidating links between genotype and phenotype in patients with rare inborn errors of immunity (IEIs) provides insights into mechanisms of immune regulation. In many autosomal dominant IEIs, however, variation in expressivity and penetrance result in complex genotype-phenotype relations, while some autosomal recessive IEIs are so rare that it is difficult to draw firm conclusions. Phenocopies arise when an environmental or non-genetic factor replicates a phenotype conferred by a specific genotype. Phenocopies can result from therapeutic antibodies or autoantibodies that target a protein to replicate aspects of the phenotype conferred by mutations in the gene encoding the same protein. Here, we consider IEIs arising from rare genetic variants in *CTLA4* and *PDCD1* and compare clinical and laboratory manifestations arising as drug-induced phenocopies (immune related adverse events, IRAEs) in cancer patients treated with immune checkpoint inhibitors (ICI) and identify outstanding questions regarding mechanism of disease.

## Introduction

Autosomal dominant loss of function mutations in *CTLA4* result in a complex syndrome of immune dysregulation and deficiency (1–3), although the syndrome is characterized by variable expressivity and incomplete penetrance. Recently, human *PDCD1* deficiency was described. So far, this syndrome appears to be exceedingly rare, whereas we have extensive experience with ICI that target PD-1 or its ligand., as well as anti-CTLA4 antibodies. Comparing and contrasting these phenocopies with IEIs of *CTLA4* and *PDCD1* might advance our understanding of the actions of CTLA4 and PD-1, and how defective expression of these molecules cause immune deficiency and dysregulation (4).

## CTLA4

CTLA4 is a transmembrane receptor that is structurally similar to CD28 but acts as an inhibitor of T cell activation (5–10). CTLA4 is expressed constitutively by regulatory T cells (Tregs) and is indispensable for immunological self-tolerance and immune homeostasis (11). Conventional T cells upregulate CTLA4 expression upon stimulation, mediated at least in part by nuclear factor of activated T-cells (NFAT) (12). CTLA4 expression has also been reported on B cells, fibroblasts, CD34^+^ stem cells and granulocytes (13–15), but the significance and action of CTLA4 expression on these cells remains to be determined. CTLA4 is expressed in immune cell malignancies including leukemic B cells and also by breast cancer cells, melanoma, and various carcinomas (16–18).

There is evidence that CTLA4 acts in several ways to modify T cell activation. CTLA4 inhibits co-stimulation by outcompeting CD28 for CD80/86 (19), and real-time competition between CTLA4 and CD28 for translocation into the central-supramolecular activation clusters (cSMAC) of immune synapses has been demonstrated (20). CTLA4 has also been reported to recruit protein phosphatase 2A (PP2A) and tyrosine phosphatase SHP-2 *via* its cytoplasmic tail, which then dephosphorylates many kinases including AKT, ERK and MEK to inhibit T cell activation (21–23). This cell-intrinsic action has been challenged, however, by reports of a mouse model expressing mutant CTLA4 lacking a cytoplasmic tail, which has no lymphocytic infiltrates or autoimmune disease (24).

Other evidence suggests that CTLA4 acts cell-extrinsically to modify immunity by reducing availability of CD80 and CD86. CTLA4 is a highly endocytic molecule and has been shown to capture and remove CD80/86 from antigen presenting cells, directing these ligands to lysosomes for degradation (25). Other possible actions include regulation of cell adhesion and motility. Ligation of CTLA4 has been postulated to increase T cell motility and reduce contact periods between T cells and antigen-presenting cells, which could prevent inappropriate activation of T cells with low-affinity for peptide-MHC complexes (26). Ligation of CTLA4 recruits PKC-eta, which forms a complex with GIT-2 and PAK-2 to modulate Treg cell-APC interactions. Consistent with this, PKC-eta deficient Tregs fail to dissociate from APCs and exhibit a defect in CD86 capture and transendocytosis (27).

## *CTLA4* Haploinsufficiency

*CTLA4* haploinsufficiency (abbreviated here as *CTLA4* ^+/-^) leads to a syndrome of immune dysregulation with a broad spectrum of clinical manifestations, and in approximately 30% of carriers, no clinical manifestations at all (1–3). In the largest cohort described to date, the median age of disease onset was 11 years, with a range of 1-59 years. Thus, in many cases, onset of clinical manifestations is not observed until adulthood. Of the clinical phenotypes, lymphoproliferation occurs frequently (73%). Autoimmune and inflammatory manifestations are also common, although there is considerable variability in the end-organs affected. Lymphocytic infiltration of lung, gastrointestinal tract, brain, bone marrow, kidney and retroperitoneal tissue have all been reported. Hematological cytopenia (immune thrombocytopenic purpura, ITP; and autoimmune hemolytic anemia, AIHA) are frequent, while atrophic gastritis, coeliac disease and pancreatitis are uncommon (1–3) (**Table 1**).

**Table 1:** Inborn errors of CTLA4 and their phenocopies.

	CTLA4 haploinsufficiency				CTLA4 checkpoint inhibitor			
**Main clinical phenotypes**		%	Immune/histological phenotype	Ref.	All (%)	Grade 3-5 (%)	Immune/histological phenotype	Ref.
	Hypogammaglobulinemia	84%	Expanded T cells with upregulated of activation markers. Increased IL-4 and IFN-γ-producing CD4^+^ T cells. Impaired suppressive function by Tregs. Lymphocytic infiltration in multiple organs.	(1–3)	ND		Dermatologic: Skin T cells infiltrate. Gastrointestinal: Enterocolitis with neutrophilic, lymphocytic infiltrate and both. Hypophysitis: Autoantibodies against TSH, FSH and ACTH-secreting cells.	
	Lymphoproliferation	73%			ND			
	Respiratory tract manifestation	57-68%			1-11%	1-2.7%		(28–30)
	Autoimmune cytopenia	62%			<1%	<1%		(31)
	Gastrointestinal manifestation	59%			30-40%	7.6-17%		(32–34)
	Dermatologic manifestation	21-56%			44-59%	1-4%		(32, 33, 35, 36)
	Thyroiditis/Hypothyroidism	14%			1.5-9%	0-1%		(32, 33)
	Liver manifestation	12%			3.8-8%	1%		(29, 32, 33)
	Hypophysitis	1%			13%	5%		(33, 37)

ND, Not Detectable.

Respiratory manifestations are common in *CTLA4* ^+/-^ patients. In addition to lymphocytic pneumonitis, many *CTLA4* ^+/-^ patients have recurrent respiratory tract infections, including pneumonia, sinusitis and otitis media. Infective complications are accompanied by hypogammaglobulinemia (84%), including reduced IgA (40%), IgG (32%) and IgM (30%) (1–3) (**Table 1**).

*CTLA4* ^+/-^ patients have hyperactivated effector T cells with increased expression of PD-1 and HLA-DR. In some studies, CD4^+^ FoxP3^+^ Treg cells have been reported to be increased (1, 3) but this has not been a consistent finding (2). B cell abnormalities in *CTLA4* ^+/-^ patients include reductions in switched memory B cells, progressive loss of all peripheral B cells, and increased CD21^low^ B cells (1–3). Interestingly, despite the hyperproliferation, T and B cells undergo increased apoptosis *in vitro* (2).

In the mouse model, *Ctla4* haploinsufficiency has not been reported to cause a phenotype but *Ctla4* ^-/-^ mice develop a lethal lymphoproliferative disorder by 3-4 weeks of age. Mice exhibit progressive skewing of the T cell compartment towards CD4^+^ T cells, lymphocytic infiltrates occur in multiple organs, and pathology is prevented by CD4^+^ T cell depletion (38). When *Ctla4* deficiency is confined to Tregs, mice exhibit delayed lymphoproliferation and fatal T cell-mediated autoimmune disease (including pulmonary lymphocytic infiltrates) by 7 weeks of age (11). T cells are activated with upregulation of activation markers CD44, CD69 and CD25 (9, 10). *Ctla4* ^-/-^ mice also exhibit macrophage and neutrophil infiltration of end-organs, including heart, lung, salivary glands, liver, bone marrow and pancreas. Interestingly, immunodeficiency observed in humans with *CTLA4* haploinsufficiency is not observed in *Ctla4* ^-/-^ mice.

## Phenocopies of *CTLA4* Deficiency

In the 1990s, Allison and colleagues identified the therapeutic potential of CTLA4 inhibition, which culminated in the development and use of ICIs targeting CTLA4 for cancer therapy (39, 40). Ipilimumab and tremelimumab bind to the same region of CTLA4 and interfere with CD80/86 recognition (41–43). Early clinical trials reported that anti-CTLA4 provided durable clinical responses and improved overall survival in a fraction of cancer patients (32, 44). ICIs are now standard of care for many forms of cancer (45). Autoimmunity and inflammatory side effects, however, emerged as significant complications in a proportion of treated patients. Severe IRAEs (Common terminology criteria for adverse events (CTCAE) severity grade of 3-5) have been reported in 19.9-24% of melanoma patients treated with ipilimumab and 52% of melanoma patients treated with tremelimumab (28, 29, 32, 35). The most common severe IRAEs are colitis, dermatitis, and endocrinopathies of hypophysitis and hypothyroidism. Hepatotoxicity, hematological cytopenia and neurologic complications are also observed but are less frequent (31–33) (**Table 1**).

Colitis of any grade, which most commonly presents with diarrhea, has been reported in 30-40% of patients treated with ipilimumab, while severe colitis/diarrhea is seen in up to 7.6-17% patients on anti-CTLA4 treatment (32, 33) (**Table 1**). Three histological types of enterocolitis are described: neutrophilic (46%), lymphocytic (15%), and combined neutrophilic and lymphocytic (38%). Neutrophilic inflammation is mainly associated with cryptitis, while lymphocytic inflammation is characterized by increased CD8^+^ T cells in the crypt epithelium and CD4^+^ T cells in the lamina propria (34). Gastrointestinal involvement is also common with *CTLA4* haploinsufficiency and histology usually reveals extensive T cells infiltration (1–3).

Severe hypophysitis is observed in 5% of melanoma patients treated with ipilimumab but is rare in *CTLA4* ^+/-^ patients (1 of 133) (3, 33) (**Table 1**). Repeated injection of anti-CTLA4 antibody results in pituitary infiltration of lymphocytes, macrophages and monocytes. In addition, pituitary autoantibodies are detected in mice and melanoma patients after injection of anti-CTLA4 antibody (37). Severe hypothyroidism has been reported in melanoma patients with anti-CTLA4 treatment but is uncommon (~1%) (33). Autoimmune thyroiditis appears to be more frequent in *CTLA4* haploinsufficiency (18 of 133) (3). Furthermore, common variants affecting the *CTLA4* promoter (49A/G or 60C/T) segregate with autoimmune hypothyroidism (46, 47).

Severe pneumonitis is observed in about 1-2% of patients treated with ipilimumab (28, 29) and bronchospasm has been reported after tremelimumab treatment (2.7%) (30). Respiratory symptoms have been reported in 57-68% patients in different studies. As noted above, many *CTLA4* ^+/-^ patients also suffer from recurrent respiratory tract infections but has been reported infrequently as a complication of anti-CTLA4 treatment (48) (**Table 1**).

Skin-related IRAEs are common and generally mild in patients receiving anti-CTLA4 treatment. Severe dermatological IRAEs, including pruritus, rash and vitiligo, are observed in up to 4% of patients treated with either ipilimumab or tremelimumab (32, 33, 35, 36). Histological analysis has revealed perivascular immune cell infiltrates in the dermis and epidermis. Although both CD4^+^ and CD8^+^ T cells are identified on biopsy of macules, CD4^+^ T cells dominate the infiltrates reported in melanoma patients receiving ipilimumab (49–51). CTLA4 blockade has also been reported to increase epidermal thickness and infiltrating T cell counts in mice with psoriasis (52). Psoriasis, atopic dermatitis and vitiligo have been observed in in 21-56% of *CTLA4* ^+/-^ patients (1, 3) (**Table 1**).

Hepatitis has been reported in 3.8-8% of patients receiving ipilimumab but severe hepatic toxicity occurs in less than 1% of patients (29, 32, 33). Liver involvement with lymphocytic infiltrate and liver failure has been observed in 12% of *CTLA4* ^+/-^ patients (1, 3).

## Comparison of CTLA4 Deficiency and Its Phenocopy

While the nature and spectrum of IRAEs after anti-CTLA4 treatment is similar to the autoimmune and inflammatory complications of *CTLA4* haploinsufficiency, these manifestations appear to be more common in *CTLA4* haploinsufficiency. This may reflect the extent of CTLA4 blockade. Consistent with this proposition, IRAEs related to ipilimumab are dose-dependent. Serious IRAEs are more common with higher doses of ipilimumab, mainly due to increased adverse events in gastrointestinal tract, skin and endocrine organs (53). Similarly, low-dose anti-CTLA4 antibody treatment in mice induces anti-parietal autoantibodies, high-dose anti-CTLA4 antibody infection leads to histologically evidence of autoimmunity (54).

Remarkably, hypophysitis is considerably more prevalent in patients treated with anti-CTLA4 antibodies than in patients with *CTLA4* haploinsufficiency. CTLA4 is expressed by both human and mice non-hematopoietic cells in the pituitary gland, and anti-CTLA4 antibodies bind to these cells. CTLA4 is also expressed by pituitary cells, particularly those responsible for secreting prolactin and TSH (37).

Differences in Fc receptor binding by ICIs have been investigated for their contributions to therapeutic actions but might also contribute to differences in IRAEs when compared with CTLA4 haploinsufficiency (55). For example, ipilimumab and tremelimumab are IgG1 and IgG2 antibodies, respectively. IgG1 binds to multiple FcRs whereas IgG2 is thought to bind to FcγRIIB and the H131 isoform of FcγRIIA. Different subclasses might also account for the longer half of tremelimumab (22 days) relative to ipilimumab (14 days) (41, 43). Different Fc components might explain other differences as well. Mouse studies have shown FcR binding results in Treg depletion by interaction with tumour-infiltrating myeloid cells, which is crucial for their anti-tumour effects (56, 57). By contrast, *CTLA4* haploinsufficiency results in deficiency of ligand binding independently of FcR ligation, which might result in differences in Treg depletion and other effects outside of the tumour environment. Differences in FcR-mediated actions might also identify pathology that is predominantly antibody-mediated. For example, hypophysitis is thought to result from complement activation by C1q binding to the Fc fragment of anti-CTLA4 antibody (37).

One major discrepancy between immune disorders in patients with *CTLA4* haploinsufficiency and those receiving anti-CTLA4 treatment is the increased susceptibility to infection in *CTLA4* ^+/-^ patients. Most *CTLA4* ^+/-^ patients present with recurrent respiratory tract infections, which is thought to result from deficiency of B cells and immunoglobulin (1, 3). Serious infections appear to be less frequent in patients receiving immune checkpoint therapy. In one study, they were observed in 54/740 patients (7.3%). Furthermore, the contribution of ICIs to infection is confounded by concurrent immunosuppression to manage IRAEs. In one study, serious infections were observed in 13.5% of melanoma patients treated with either corticosteroids or infliximab but in only 2% in those who did not require immunosuppression (48).

Since LRBA competes with AP-1 for the YVKM motif on the cytoplasmic tail of CTLA4 to protect CTLA4 from lysosomal degradation (58), LRBA deficiency results in reduced CTLA4 expression and could therefore be informative for understanding the *CTLA4* ^+/-^ phenotype. Similar to *CTLA4* ^+/-^ patients, *LRBA* deficiency also confers increased risk of recurrent respiratory tract infections, and most patients with homozygous or biallelic mutations in *LRBA* are also diagnosed in early childhood with hypogammaglobulinemia (57%), B cell lymphopenia, particularly affecting memory B cells and plasmablasts (58–60), while heterozygous carriers are healthy. Most *LRBA* ^-/-^ patients have immune dysregulation encompassing enteropathy and hematological cytopenia (AIHA and ITP). Organomegaly, including splenomegaly and lymphadenopathy, is also prevalent, while T1D and hepatitis are less common (58–60). Two groups have reported that *Lrba* ^-/-^ mice do not have any sign of immunological disorders, either at steady state or after challenge with virus or bacterial infection. Another group reported that *Lrba* ^-/-^ mice are susceptible to DSS-induced colitis, although this phenotype was suggested to arise from dysregulation of TLR signaling rather than impaired CTLA4 expression (61–63). In mice, conventional B and T cell development does not appear to be affected by LRBA deletion, although peritoneal B1-a cells are reduced.

## PD-1

In the 1990s, Honjo and colleagues discovered and characterised PD-1 as a negative T cell regulator (64–67). The therapeutic potential of PD-1 blockade in cancer therapy was illustrated in *Pdcd1* ^-/-^ mice and then confirmed in cancer patients after anti-PD-L1 treatment (68). PD-1 is expressed by T cells, NK cells, B cells and activated monocytes (69). PD-1 expression is considered to be a marker of cell exhaustion, and PD-1^+^ cells exhibit reduced cytokine production and reduced proliferative capacity (70–72). Nuclear factor of activated T cells, cytoplasmic 1 (NFATc1) and interferon-stimulated responsive element (ISRE) bind directly to the PD-1 promoter to upregulate PD-1 expression in response to TCR and IFN-α stimulation, respectively (73, 74), whereas T-bet and Blimp-1 suppress PD-1 expression (75, 76). Posttranslational modifications also regulate PD-1 expression, which provides potential novel avenues for PD-1-related therapy. Fucosylation of PD-1 at positions N49 and N74 by Fut8, a core fucosyltransferase, is required for cell-surface expression of PD-1 (77). Moreover, PD-1 is degraded in proteasome after Lys48-linked poly-ubiquitination by the E3 ubiquitin ligase, FBXO38. Deletion of FBXO38 leads to faster tumor progression with increased PD-1 expression in tumor-infiltrating T cells (78).

There are two PD-1 ligands. PD-L1 is expressed on T cells, B cells, dendritic cells, macrophages and non-hematopoietic cells, while PD-L2 is restricted to dendritic cells and macrophages (69). PD-L1 expression is upregulated in many tumors, including melanomas, non-small cell lung cancer and ovarian cancer (79–82). Upon IFN-γ stimulation, PD-L1 upregulation is mediated by various transcription factors, including IRF-1, MyD88, TRAF6 and MEK (83, 84). In addition, chimeric nucleophosmin (NPM) and anaplastic lymphoma kinase (ALK) induce the expression of STAT3, which upregulates PD-L1 expression (85). PD-L1 expression is also regulated post-transcriptionally. Glycogen synthase kinase 3β (GSK 3β) phosphorylates non-glycosylated PD-L1, leading to the proteasomal degradation by β-transducin repeat-containing protein (β-TrCP) (86), while COP9 signalosome 5 (CSN5) induced by NF-κB p65 (RelA) upon TNF-α stimulation inhibit ubiquitination and degradation of PD-L1 (87).

Ligation of PD-1 by PD-L1 results in transduction of a negative signal that suppresses T cell activation, cytokine production, survival and proliferation. LCK phosphorylates the immunoreceptor tyrosine-based switch motif of PD-1 cytoplasmic tail (88), recruiting Src homology region 2 domain containing phosphatase-1 (SHP-1) and SHP-2, which dephosphorylate many signaling molecules, including ZAP70/CD3zeta and ERK in TCR signaling pathway (88, 89), as well as PI3K/AKT/mTOR in CD28 signaling pathway (90–92). There is evidence that PD-1 binds preferentially to SHP-2 and dephosphorylates the CD28 cluster in the immune synapse (93). Additionally, ICOS co-stimulation of T cells for proliferation and cytokine production is also inhibited by PD-1 ligation (94). PD-1 also suppresses TCR-driven signal that stops cellular migration to increase the contact time of antigen-specific T cells with dendritic cells (70). The inhibitory function mediated by PD-1-PD-L1 ligation could also be indirect. In the absence of PD-1 ligation, TCR stimulation upregulates the expression of Ser/Thr protein kinase CK2, which stabilizes PTEN protein as a negative regulator of PI3K/Akt signaling pathway (95).

## *PDCD1* Deficiency

Recently, a rare, homozygous frameshift mutation (c.105dupC, p.T36Hfs*70) in *PDCD1* was identified in a patient with tuberculosis and autoimmunity (96). The mutation was shown to abrogate PD-1 expression. The patient was diagnosed with type 1 diabetes (T1D), hypothyroidism and juvenile idiopathic arthritis (JIA) by the age of 3 years, developed large, multifocal intraperitoneal abscesses and abdominal tuberculosis by the age of 10 years, and died of pneumonitis one year later. Stimulated leukocytes from the patient exhibited reduced IFN-γ production. The numbers of Vδ2^+^ γδ T cells, mucosal-associated invariant T cells, and CD56^bright^ natural killer cells were decreased, but helper T cell subsets were within normal range. Interestingly, the patient exhibited hepatosplenomegaly with expanded CD38^+^ activated and RORγT^+^ CD4^-^ CD8^-^ double-negative αβ T cells, similar to the phenotype displayed by *STAT3* GOF patients, and STAT3-dependent cytokines IL-6 and IL-23 were increased (96) (**Table 2**).

**Table 2:** Inborn errors of PDCD1 and their phenocopies.

	Genetic PD-1 deficiency				PD-1 or PD-L1 immune checkpoint inhibitor			
**Main clinical phenotypes**		%	Immune/histological phenotype	Ref.	All (%)	Grade 3-5(%)	Immune/histological phenotype	Ref.
	Dermatologic manifestation	Present*	Impaired IFN-γ production by T cells. Decreased Vδ2+ γδ T cells, mucosal-associated invariant T cells, and CD56bright natural killer cells. Increased CD4- CD8- double-negative αβ T cells.	(96)	16-50%	<3%	Dermatologic: Increased serum IL-6 and skin CD8/CD4 ratio. Gastrointestinal: Neutrophilic and lymphocytic inflammation. Liver: lymphocyte infiltration and rare plasma cell and eosinophil.	(28, 97–100)
	Thyroid diseases	Present			8-24.5%	<1%		(28, 98, 101–105)
	Gastrointestinal manifestation	Not described			1-20%	1-2%		(98, 102, 105–107)
	Respiratory tract manifestation	Present			2.2-16%	1-2%		(98, 101, 102, 105)
	Liver manifestation	Present			5.2-18%	<1%		(98, 103, 108)
	Type 1 diabetes	Present			<1%	<1%		(28, 101, 104)
	Tuberculosis	Present			–			–

* - Only one child with inherited PD-1 deficiency is described yet.

About one third of *Pdcd1* ^-/-^ C57BL/6 mice develop arthritis and mild proliferative glomerulonephritis by 6 months of age, with extensive renal IgG3 and C3 deposition, and these manifestations become more frequent and severe over time (65). On a BALB/c background, only 30% of *Pdcd1* ^-/-^ mice survive to 40 weeks due to autoimmune myocarditis (66).

## Phenocopies of *PDCD1* Deficiency

IRAEs are less frequent in patients receiving ICIs directed against PD-1 and PD-L1 than with CTLA4 ICIs. Severe IRAEs are have been reported in up to 16.3% of melanoma patients and 10% of non-small-cell lung cancer patients treated with nivolumab (anti-PD-1) (97, 106, 107, 109, 110), 10.1-14.7% of melanoma patients and 9.5-26.6% of non-small-cell lung cancer patients treated with pembrolizumab (anti-PD-1) (28, 101–104), and 11-15% of non-small-cell lung cancer or metastatic urothelial cancer patients with atezolizumab (anti-PD-L1) (105, 111, 112). By contrast with IRAEs after ipilimumab, which appear to be dose-dependent, IRAEs with anti-PD-1/PD-L1 are independent of dose (53, 102, 113). The IRAEs related to anti-PD-1/PD-L1 treatment are mostly mild (28, 97, 105, 107, 110). Pembrolizumab and nivolumab bind to partially overlapping epitopes on PD-1, and both outcompete PD-L1 for binding to PD-1 due to their high affinity. Interestingly, both agents are human IgG4 antibodies, in which Fc regions exhibit low affinity for complement protein C1q and FcRs (114, 115).

Severe colitis has only been reported in 1-2% patients after anti-PD-1/PD-L1 treatment, which is much less frequent than after anti-CTLA4 therapy (98, 102, 105–107) (**Table 2**). Histology reveals both neutrophilic and lymphocytic inflammation (116). Respiratory IRAEs have been reported in 2.2-16% of patients after anti-PD-1/PD-L1 treatment. Severe pneumonitis was reported in 0.8-2% of patients treated with pembrolizumab (98, 101, 102, 105) (**Table 2**). Anti-PD-1/PD-L1 therapy related pneumonitis is more likely to occur in non-small cell lung cancer than melanoma and renal cell carcinoma (117). Cryptogenic organizing pneumonia is the main pattern in PD-1 inhibitor-related pneumonia, followed by nonspecific interstitial pneumonia (118).

Thyroiditis can present with either hypothyroidism or hyperthyroidism, and is observed in 8-24.5% patients after anti-PD-1/PD-L1 treatment. Severe hypothyroidism or hyperthyroidism occurs in less than 1% (28, 98, 101–105). Interestingly, individuals with pre-existing anti-thyroid autoantibodies are significantly more susceptible to thyroid IRAEs induced by PD-1 inhibitor (119, 120). T1D and adrenal insufficiency have been observed in about 1% of patients with pembrolizumab treatment (28, 101, 104) (**Table 2**).

Rash, pruritus and vitiligo occur in 16-50% of patients, while severe skin disorders occur in less than 3% (28, 97, 98) (**Table 2**). Patients with pre-existing autoantibodies and rheumatoid factor are more susceptible to skin IRAEs (99). In patients with pre-existing psoriasis, anti-PD-1 treatment has been shown to increase CD8^+^/CD4^+^ T cells ratio of infiltrating skin lymphocytes. The level of IL-6 but not IL-17A, IFN-γ and IL-8 in serum is significantly increased in cancer patients developing psoriasis-like dermatitis after anti-PD-1 treatment (100). Severe transaminitis occurs in 1% of patients after anti-PD-1 treatment (98, 103). Histology analysis indicates most patients exhibit the lobular inflammation with lymphocytic infiltrate and rare infiltration of plasma cells and eosinophils (108).

## Comparison of *PDCD1* Deficiency and Its Phenocopies

The spectrum and manifestations of IEIs of *PDCD1* and phenocopies arising as IRAEs after PD-1 inhibitors overlap. *PDCD1* deficiency (c.105dupC, T36Hfs*70) resulted in T1D and hypothyroidism at the age of 3 years, rash and stomatitis at the age of 11 years (96). A proteome-wide serum autoantibody profile revealed antibodies related to autoimmune thyroiditis and T1D (96).

Experimental models provide insight into these complications. First, PD-1-PD-L1 is critical to maintain intestinal tolerance and prevent experimental autoimmune enteritis. In a transgenic mouse model in which ovalbumin (OVA) was expressed as a neo-self-antigen by intestinal epithelial cells, either PD-L1 deletion and blockade resulted in significant weight loss and intestinal inflammation in mice transferred with OVA-specific CD8^+^ T cells (121). Similarly, in a model of intestinal injury, *PD-L1* ^-/-^ mice exhibited increased mortality and weight loss, diarrhea and rectal bleeding. PD-L1 expression on non-hematopoietic intestinal parenchyma prevented TNF-α production and conferred protection from intestinal inflammation. Interestingly, *PD-L1* ^-/-^ *Rag* ^-/-^ mice have a significantly higher death rate and morbidity than *Rag* ^-/-^ mice, indicating a contribution by innate immunity (122). Interestingly, however, gastrointestinal abnormalities were not reported in the patient with *PDCD1* deficiency (96).

By contrast, destructive thyroiditis was observed in both the *PDCD1*-deficient patient and in cancer patients treated with anti-PD-1 antibodies. Furthermore, thyroid infiltration of PD-1^+^ T cells is observed in sporadic Graves’ disease (123), and a mouse thyroiditis model induced by thyroglobulin immunization is exaggerated by anti-PD-1 treatment, which is prevented bydeletion of CD4^+^ T cells. In this model, thyroid infiltrating CD4^+^ T cells acquire cytotoxic features and potentially kill thyrocytes *via* specific recognition of thyroglobulin antigen (120).

T1D is observed in cancer patients after anti-PD-1 treatment and occurred in the *PDCD1*- deficient patient. A 7146G/A polymorphism in *PDCD1* gene has been reported to confer significantly increased susceptibility to T1D (124). T1D is accelerated and completely penetrant after PD-1 deletion in NOD mice, which appears to result in enhanced T cells infiltration of β-islets, with increased IFN-γ production (125) although autoantibodies against insulin were not increased compared with WT NOD mice (125). Deficiencies of *PD-L1*, *PD-L* ^-^ and *PD-L1* have all been shown to accelerate development of diabetes in NOD mice. Pancreatic lymph nodes from *Pdl1* ^-/-^ *Pdl2* ^-/-^ NOD mice also have more IFN-γ and TNF-α producing T cells. Interestingly, PD-L1/PD-L2 expression on nonlymphoid cells is sufficient to control the progression of autoimmune diabetes (126).

Finally, the *PDCD1* patient had dermatitis (96). Consistently, mice with *Pdcd1* deletion on CD8^+^ T cells are more susceptible to psoriasis-like dermatitis induced by imiquimod (R848, a toll-like receptor 7/8 agonist), which is ameliorated by anti-IL-6 receptor blockade (100). In another contact hypersensitivity mouse model induced by hapten, PD-1 deletion and anti-PD-L1 treatment also lead to enhanced skin infiltration by CD8^+^ T cells (127).

## Conclusions

IRAEs observed in patients treated with antibodies targeted at CTLA4 and PD-1 and the receptors of their ligands phenocopy the autoimmune manifestations of patients with IEIs of *CTLA4* and *PDCD1*. There are differences in the spectrum of autoimmune manifestations between IEIs and IRAEs, such as hypophysitis. ICIs phenocopy defects in ligand binding, but also result in FcR ligation. This difference merits further investigation as a possible cause of phenotypic discrepancies between IEIs and their phenocopies. Another important observation, however, is that analysis of IEIs and their therapeutic phenocopies suggest that humans are more dependent on checkpoint inhibition than mice for protection against autoimmunity and inflammation.

Clinical manifestations arise in the majority of patients with *CTLA4* haploinsufficiency. By contrast, lymphocytic pathology arises only in mice homozygous for *Ctla4* deletion. This suggests that in addition to the genetic defect in *CTLA4* in its IEI, or after CTLA4 blockade, additional factors may promote inflammatory pathology in humans that do not act in the mouse model, at least not in the specific pathogen free environments in which experimental mice are maintained. This conclusion is supported by observations with IEIs arising from *LRBA* deficiency, which results in a reduction in CTLA4 expression. LRBA deficiency often results limited autoimmunity in humans, whereas *Lrba*-deficient mice are either healthy, or exhibit inflammation only after substantial environmental stress (e.g. DSS administration), which might provide further evidence that humans are more sensitive to changes in CTLA4 expression than mice.

The other significant phenotypic discrepancy is immune deficiency. These are common in IEI affecting CTLA4, but are not observed as IRAEs, nor do they feature in mouse models of *Ctla4* deficiency. *LRBA* mutations also result in hypogammaglobulinaemia and B cell deficiencies. Based on the distribution of CTLA4, LRBA and PD-1 expression, the effect is unlikely to be cell-intrinsic to the B cell compartment, but this will need to be resolved empirically. In some patients, CD21^low^ B cells, which are said to be exhausted, have been observed to be expanded in patients with *CTLA4* deficiency, and this population has been simultaneously used to explain the hypogammaglobulinemia, and the increased incidence of autoantibodies (128, 129). While antibody deficiency has been observed in humans with *LRBA* deficiency, and this might reinforce the evidence that *CTLA4* deficiency causes antibody deficiency, other potential mechanisms, most notably B cell intrinsic defects in autophagy could explain this phenotype.

Further work will be required to resolve this fascinating discrepancy. Possible contributions include the age of onset of the defect in immune regulation, which is of course congenital with IEIs abut is often only encountered much later with ICIs. The magnitude of inhibition may also be important, since, as described above, there is a dose-response effect of observed with CTLA4 inhibition. Finally, it is plausible that microbiological challenge from infection may exacerbate the phenotype in humans, and that this could be less apparent in mice maintained under specific pathogen-free conditions.

## Author Contributions

Both authors contributed to the conceptualization and drafting of the manuscript. All authors contributed to the article and approved the submitted version.

## Funding

National Health and Medical Research Council, Australia, GNT1113577 Cancer Australia, GNT1130330.

## Conflict of Interest

The authors declare that the research was conducted in the absence of any commercial or financial relationships that could be construed as a potential conflict of interest.

## Publisher’s Note

All claims expressed in this article are solely those of the authors and do not necessarily represent those of their affiliated organizations, or those of the publisher, the editors and the reviewers. Any product that may be evaluated in this article, or claim that may be made by its manufacturer, is not guaranteed or endorsed by the publisher.
